# Knowledge gaps and areas for synthesis in experimental studies of context‐dependent predation risk

**DOI:** 10.1002/ecy.70493

**Published:** 2026-09-08

**Authors:** Nicole E. Peckham, Scott D. Peacor, Michael J. Sheriff, Justine A. Smith, Nathan J. Dorn, Michael J. Cherry, Tanya L. Rogers, David L. Kimbro

**Affiliations:** ^1^ Department of Marine and Environmental Science Northeastern University Nahant Massachusetts USA; ^2^ Department of Fisheries and Wildlife Michigan State University East Lansing Michigan USA; ^3^ Biology Department University of Massachusetts Dartmouth North Dartmouth Massachusetts USA; ^4^ Department of Wildlife, Fish, and Conservation Biology University of California–Davis Davis California USA; ^5^ Department of Biological Sciences and Institute of Environment Florida International University Miami Florida USA; ^6^ Caesar Kleberg Wildlife Research Institute Texas A&M University‐Kingsville Kingsville Texas USA; ^7^ Southwest Fisheries Science Center National Oceanic and Atmospheric Administration Santa Cruz California USA

**Keywords:** context‐dependence, ecology of fear, literature review, nonconsumptive effects, non‐lethal, trait response, trait‐mediated indirect effects

## Abstract

A growing number of experimental studies address whether the anti‐predator trait responses of prey are associated with costs to prey fitness (non‐consumptive effects) or to the fitness of other species (trait‐mediated indirect effects). Because ecologists remain keen to determine when these risk effects matter, there is a need to know how much of this literature has also evaluated the context‐dependence of risk effects. Such knowledge would identify both understudied research directions and richly investigated areas whose synthesis could advance general understanding of predation risk effects. As a guide for ecologists, we reviewed publications from 1990 to 2021 that experimentally manipulated both predation risk (presence/absence of risk) and another aspect of biotic or abiotic condition (context). We classified each experiment according to a common scheme: the manipulated context was assigned to a Focus (predator, prey, resource, or environment) and Category (community, population, or individual for biotic contexts; temperature, water, habitat, or anthropogenic disturbance for abiotic contexts). A finer categorization of Specific Context (e.g., predator identity, resource abundance) was assigned within each Focus and Category. We also identified the System (marine, freshwater, terrestrial) and Venue (laboratory or field) of each experiment. We classified 433 manipulations (339 publications) falling within 49 different Specific Contexts. Many experiments (63%) manipulated aspects of the predator and/or prey Foci, independently and factorially. Additionally, several Specific Contexts have many published papers and could be suitable for synthesis, including predator and prey identity, predator diversity, prey and resource abundance, habitat structure, and temperature. Meanwhile, manipulations of resources of the prey are relatively lacking. Because most experiments (51%) featured freshwater systems, more marine and terrestrial experiments are needed. Regardless, an imbalance between laboratory (69%) and field (31%) experiments highlights the need for more field investigations. We discuss potential contributors to biases in the literature and consider how understudied contexts should be prioritized. Consequently, our review undergirds future work on the context‐dependency of predation risk effects. As many biotic and abiotic contexts are impacted by anthropogenic activities, optimizing the integration of experimental approaches with other research efforts is crucial to furthering the development and application of predation risk theory.

## INTRODUCTION

Since the classic publication by Lima and Dill (1990) highlighted the importance of studying how prey alter their traits to reduce the risk of being eaten by predators, the study of predation risk has grown rapidly (Peacor et al., 2022). In three decades, over 4000 publications have examined how predation risk alters the behavioral, morphological, and physiological traits of prey. An important fraction (9%) of those publications also addressed whether the anti‐predator trait responses of prey are associated with costs to prey fitness (nonconsumptive effects, NCEs) or to the fitness of other species, such as prey resources or competitors (trait‐mediated indirect effects, TMIEs; Peacor et al., 2020). Because theory and logical arguments suggest that NCEs and TMIEs could be as or even more important than the consumptive effects of predators (Abrams, 2008; Peckarsky et al., 2008; Werner & Peacor, 2003), ecologists remain keen to determine when risk effects matter and how they operate in nature (Peacor et al., 2022).

We suspect a significant yet unknown amount of this published research has explicitly focused on understanding the context‐dependency (Chamberlain et al., 2014) of predation risk effects, specifically through orthogonal experimental manipulations. ‘Context‐dependent predation risk’ refers to effects of risk that depend on biotic or abiotic conditions (context), such as resource abundance, temperature, or predator hunting mode. For example, in old field systems, actively hunting versus sit‐and‐wait predatory spiders (i.e., predator identity context) induced different behavioral responses in grasshopper prey and consequently, directionally opposite TMIEs on plant diversity and nutrient cycling (Schmitz, 2008). As published experiments have accumulated on contexts, meta‐analytic reviews have examined effects of predator identity (Preisser et al., 2007), resource supply (Preisser et al., 2009), habitat refugia (Orrock et al., 2013), and prey abundance (Bolnick & Preisser, 2005) to identify emergent trends (but see Peacor et al., 2025 who raise methodological concerns).

In recent literature reviews, Peacor et al. (2022) encountered many additional contexts pertaining to predators (e.g., diversity, abundance, diet) and the environment (temperature, acidification), and Wirsing et al. (2021) developed conceptual models with testable predictions about the effect of several contexts. For example, the energetic/satiation state of a predator is predicted to lead to oscillating NCE strengths: sit‐and‐wait predator behavior during high energy/satiation and actively roaming predator behavior during low energy states are predicted to cause stronger and weaker NCEs on prey, respectively. Wirsing et al. (2021) also highlighted the need for research to test the influence of multiple interacting contexts. There is currently a need to organize the rich literature on context‐dependence to determine where previous syntheses can be extended, what new syntheses can be conducted (and hypotheses tested), and what contexts remain understudied. For instance, it is unknown whether sufficient publications exist to examine variation among freshwater, marine, and terrestrial systems, which can vary in the strength of indirect predator effects (Shurin et al., 2002) and the trophic distribution of biomass (Bar‐On et al., 2018). Given a limited research funding environment and a multitude of contexts shifting with climate change, diversity loss, and biological invasion, we suggest that introspection is needed so that the study of predation risk can take its next step forward strategically and efficiently.

To initiate such introspection, we examined 31 years (1990–2021) of literature on predation risk and identified all experimental studies that manipulated predation risk (presence/absence of risk) along with another aspect of biotic or abiotic conditions (context). We specifically focused on experiments (as opposed to observational and modeling studies) as a more organized experimental literature could better motivate future studies, help interpret previous observational research, and test theory. We organized these studies according to a framework we developed inspired by the categorizations used in Wirsing et al. (2021). Based on our categorizations, we highlight contexts potentially suitable for synthesis (i.e., those with many studies) and for testing cross‐system variation (freshwater, marine, and terrestrial). In addition to highlighting how many experiments manipulated multiple contexts, we also partitioned the literature into laboratory and field experiments, as outcomes may differ between laboratory and field settings within the same system (Stachowicz et al., 2008; Werner, 1998). Equally important, we used the summarized categorizations to identify contexts with little published research. The large number of understudied contexts prompted us to probe the database for explanations underlying the imbalance and to consider how understudied contexts should be prioritized. Consequently, our review initiates and undergirds future work on the context‐dependency of predation risk effects.

## METHODS

### Review of experiments on context‐dependent risk effects

The starting point for our review was a database assembled by Peacor et al. (2022), which synthesized the literature of predation risk effects from 1990 to 2018. That database was assembled using three sub‐searches to comprehensively identify all studies (observational, experimental, and modeling) that examined NCEs and/or TMIEs. In the first sub‐search, the most prominent risk‐effect terms (see online appendix in Peacor et al., 2022) were searched in Web of Science (All Databases), which yielded 4013 potentially relevant publications. After removing papers whose titles and abstracts focused on non‐ecological topics, two researchers independently scored each of the remaining papers for relevance using a decision tree (Peacor et al., 2022). When the two reviewers failed to reach consensus, the lead author (SDP) provided a third evaluation, often after consultation with the original authors of the publication. The second sub‐search identified all papers that referenced 10 classic and highly cited papers about risk effects (online appendix, Peacor et al., 2022). After removing duplicates with the first sub‐search, an additional 2358 papers were scored. A third sub‐search was conducted on the 3891 papers that cited the classic publication by Lima and Dill (1990). Due to this large number, Peacor et al. (2022) randomly selected and reviewed 330 citing papers. Although most of these papers concerned predation risk effects, only 4% of the publications quantified fitness consequences of trait‐responses to risk (i.e., NCEs, TMIEs) and were missed by the first two sub‐searches. When we reviewed those missing papers, we found that 2% (*n* = 7) of the publications missed by the first two sub‐searches concerned experiments on the context‐dependency of predation risk. Consequently, some relevant papers without risk‐effect terms in their titles, abstracts, and key words are missing from the current review. Using the same methods as Peacor et al. (2022), we expanded the database to include the years 2019 and 2020.

To examine the scope of the literature on the context‐dependency of predation risk, we filtered the expanded database to retain only publications that factorially manipulated predation risk (presence/absence of risk) along with some aspect of biotic or abiotic conditions (context). A qualifying example would be a study with two levels of predation risk (present, absent) and two levels of temperature (ambient, elevated) with all four treatment combinations. While not technically representing a fully orthogonal design of manipulated factors, we did include comparative experimental approach (CEA) studies (2.5% of final database) with repeated experiments of presence/absence of risk across quantified spatial gradients (low to high) of an environmental context (Menge et al., 2003). The context manipulated in each experiment was then categorized as described below. Studies measured a wide range of response variables (e.g. growth, fecundity, mortality), but we classified experiments based only on the experimental design, not on the specific response variables measured.

### Categorization of experiments

Our categorization framework was organized around four Foci indicating which properties of the system were manipulated in addition to the presence/absence of predation risk: predator (source of risk, encompassing top‐down control), prey (organism responding to risk), resource (trophic level below prey, encompassing bottom‐up control), or environment (physical conditions). These Foci have parallels with the categorizations used by Wirsing et al. (2021), which identified properties of the ‘predator’ (e.g., size), ‘prey’ (e.g., energetic state), and ‘setting’ (e.g., refugia) that alter the perception of and behavioral response of prey to risk. However, the studies we consider are not limited to those examining behavioral responses, and we distinguish ‘settings’ associated with trophic resources used by the prey and the physical environment.

**FIGURE 1:**
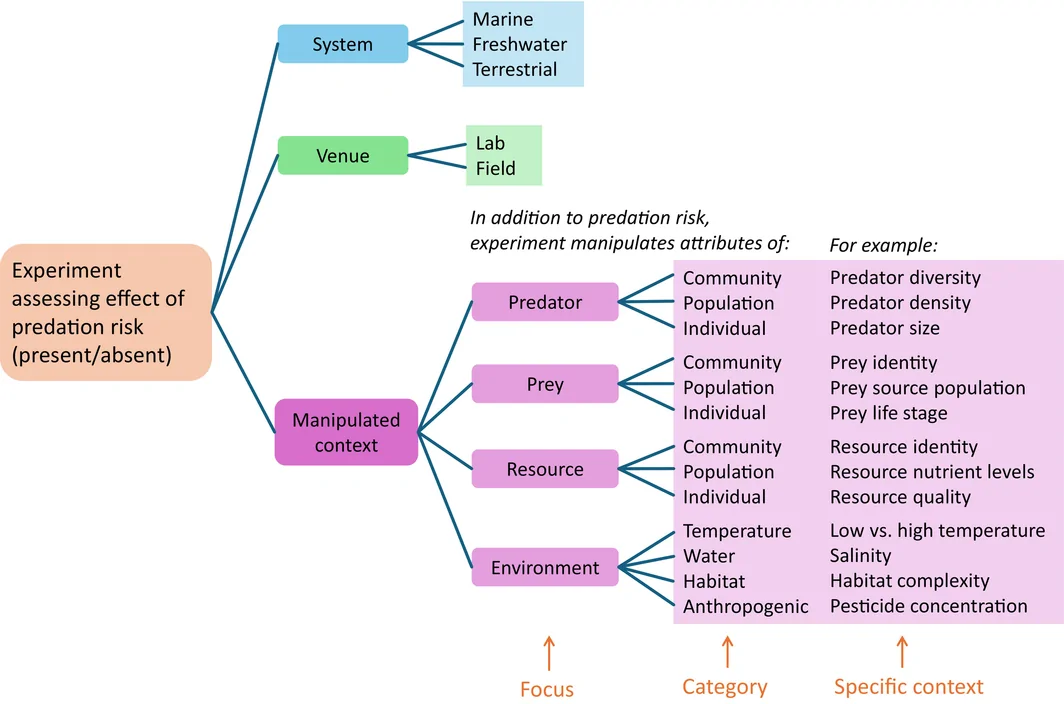
Diagram of categorizations applied to each experiment assessing the context dependency of predation risk. Each experiment was assigned to a System (marine, freshwater, or terrestrial) and Venue (lab or field). The context manipulated by the experiment was assigned to a Focus (predator, prey, resource, or environment) and a Category based on the attributes of the predator–prey system that were manipulated in the experiment (e.g., study manipulated attributes of the predator community). Examples of Specific Contexts manipulated within each Focus and Category are also given (e.g., predator diversity).

For each published experiment, we categorized the manipulated context into one of the four Foci depending on which ecological attributes were manipulated: predator, prey, resource, or environment (Figure 1). Within the manipulated biotic Foci (predator, prey, resource), we sorted experiments into three Categories reflecting different scales of biological organization: ‘community’ experiments manipulated *multiple* species (e.g., monoculture vs. polyculture diversity treatments), ‘population’ experiments manipulated the abundance or size‐structure of a *single* species, and ‘individual’ experiments manipulated an *individual* organism's state (e.g., size or energetic state). Within the environment Focus, experiments were sorted into four Categories: ‘temperature’, ‘water’, ‘habitat’, and ‘anthropogenic disturbance’. Within each Focus and Category, we further classified each experiment into a Specific Context that was manipulated (e.g., ‘identity’ and ‘diversity’ are two different Specific Contexts within ‘predator community’). See Appendix S1: Table S1 for descriptions of each Specific Context. Finally, for each experiment (independently of the manipulated context), we identified the System type in which it was conducted (freshwater, marine, terrestrial) and the Venue (field or lab). The hierarchical scheme is presented in Figure 1.

If a publication manipulated multiple contexts with and without predation risk, we also recorded whether the multiple contexts were manipulated independently of one another or whether they were factorially crossed (including all treatment combinations of both manipulated contexts and predation risk). In some experiments, the risk factor was intentionally expanded in an ordinal manner to test the influence of Specific Contexts such as predator identity, predator diversity, and predator density. In these cases, we considered the levels of the predation risk factor to represent a Specific Context within the predator Focus. Finally, for some Specific Contexts, researchers used a diversity of methods to manipulate different attributes of the context. For instance, experiments that increased the average water salinity, the variance of water salinity, or the minimum of water salinity were all categorized into the Specific Context of environment/water/salinity. Similarly, experiments that brushed an individual plant leaf or sprayed it with jasmonic acid were all grouped into the Specific Context of resource/individual/inducible defense.

### Highly versus understudied Specific Contexts

After noticing disparities in the representation of different Specific Contexts in the literature, we developed and tested two hypotheses for why some contexts might be more highly represented than others. First, because ecological theory can drive empirical work, we hypothesized that the Specific Contexts with more publications would be better motivated by theory than Specific Contexts with fewer publications. This hypothesis was tested by randomly drawing publications from each of the seven highly studied Specific Contexts (publication *n* ≥ 20) and from the collection of understudied Specific Contexts (*n* < 5), and assigning them a theory score (detailed below). Initially, a minimum of 25% of publications from each of the seven highly studied Specific Contexts and from the collective 29 understudied Specific Contexts were randomly selected to receive a theory score to ensure adequate sampling coverage. An additional 80 studies were randomly drawn to increase sample size, resulting in an average of 53% of publication assessment from each highly studied Specific Context and 49% publication assessment from the 29 understudied Specific Contexts.

For each paper, we scored its connectedness to theory on a scale from 0 to 4. A score of 0 indicates that the Introduction of the publication lacked any mention of a theory or hypothesis as motivation for the manipulated context. In contrast, a score of 4 indicates that the Introduction developed a testable hypothesis about the context based on a model developed for the particular publication, with model parameters based on the experimental results from the featured study system. See Appendix S1: Table S2 for further details on theoretical connectedness scoring. After scoring the publications, we calculated an average theory score for the selected papers within each of the highly (7) and understudied (29) Specific Contexts. Next, we used Pearson correlation analysis to test for a significant relationship between average score of theoretical connectedness and publication number (log transformed to improve normality and homoscedasticity). We also present boxplots of the theoretical connection scores for publications within each Specific Context.

A second testable hypothesis was that certain authors or groups of co‐authors may account for much of the productivity in a Specific Context. To test this second hypothesis, we identified researchers within each of the seven highly studied Specific Contexts that published two or more publications. In this subset of the literature, publications were grouped by any level of shared co‐authorship from one co‐author to multiple, identical co‐authors. Typically, a single author name resulted in a fused group of publications with multiple, non‐identical co‐authors. Next, we tallied the number of publications produced by each co‐author group and divided their collective publication number by the total number of publications on that Specific Context. Across all seven of the highly studied Specific Contexts, we also calculated the proportion of publications that were collectively produced by all the co‐author groups.

## RESULTS

From 1990 to 2021, there were 602 publications about predation risk effects that quantified fitness costs to prey (NCEs) or to other species (TMIEs). Of those, 339 publications experimentally investigated the context‐dependency of predation risk effects, manipulating biotic or abiotic conditions along with predation risk presence/absence. These publications contained a total of 433 context manipulations that were assigned to 49 different Specific Contexts (Table 1).

**TABLE 1:** Summary of literature review results from 339 publications, giving the number of context manipulations, number of experiments, and number of Specific Contexts explored within each Focus and Category. Specific Contexts with ≥20 different experiments (ready for synthesis) and with 5–19 different experiments (nearly ready for synthesis) are also given within each Focus and Category.

Focus	Category	No. context manipulations	No. Specific contexts	Specific contexts ≥20 experiments	Specific contexts 5–19 experiments
All	All	433	49		
Predator	All	129	14		
Prey	All	143	21		
Resource	All	62	10		
Environmental	All	99	19		
Predator	Community	90	5	Diversity, identity	Guild complexity, interspecific lethal
Predator	Population	19	7		Abundance
Predator	Individual	28	4		Diet, exposure
Prey	Community	50	4	Identity	Diversity, interspecific competition
Prey	Population	86	12	Abundance	Age, culling, infection, prior risk exposure, source
Prey	Individual	17	9		Age
Resource	Community	13	4		Identity
Resource	Population	47	6	Abundance	Quality
Resource	Individual	2	2		
Environmental	Temperature	33	5	Temperature	
Environmental	Water	13	7		
Environmental	Habitat	29	5	Structure	
Environmental	Anthropogenic	27	4		Runoff pollutant

**FIGURE 2:**
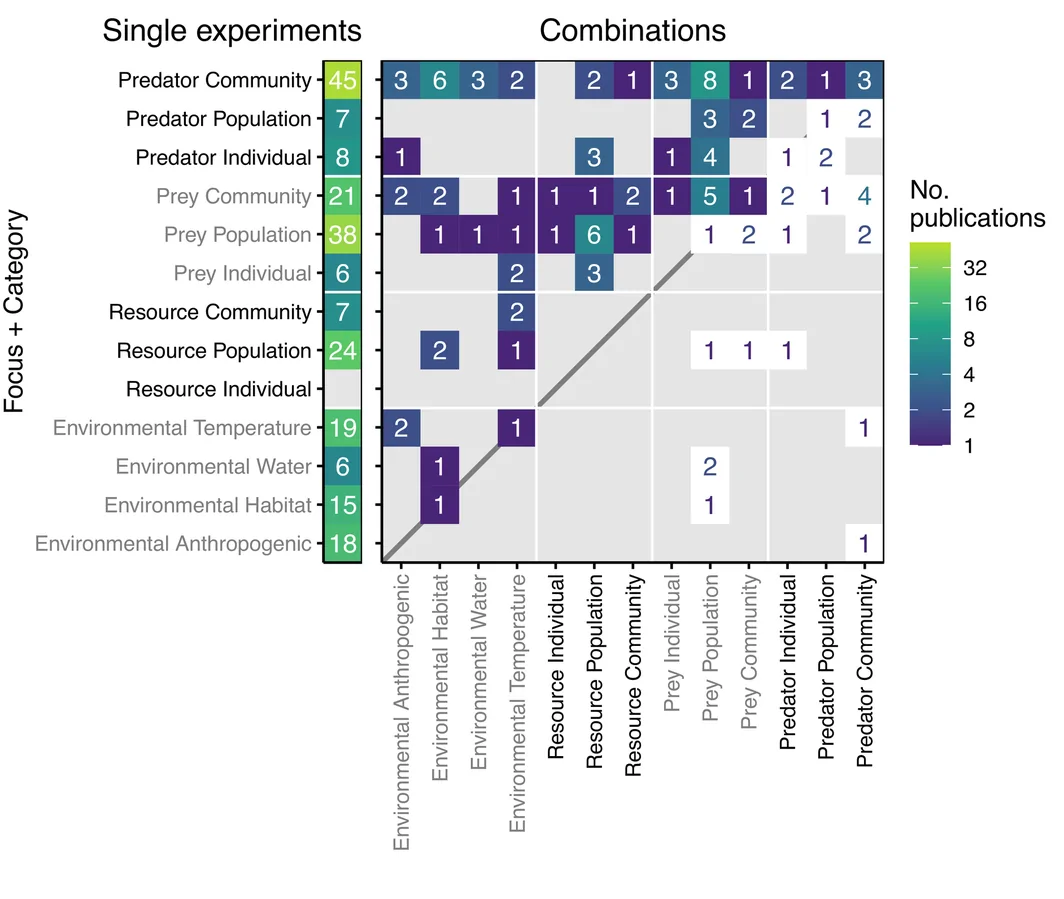
Matrix of publications that manipulated predation risk in combination with a single or multiple contexts within each Focus and Category. Publications in the left column conducted a single experiment. Publications in the matrix conducted multiple experiments with manipulated contexts either factorially crossed or studied independently. Those on the diagonal conducted two or more experiments within a Focus and Category. Those on the off‐diagonal conducted experiments in two different Focus/Categories. Those with colored backgrounds (above diagonal) examined interactions between the different Focus/Categories through a factorial experimental design. Those with white backgrounds (below diagonal) examined the different Focus/Categories independently of one another. The matrix includes 11 publications that examined three Focus/Categories (the additional Focus/Categories are not shown).

**FIGURE 3:**
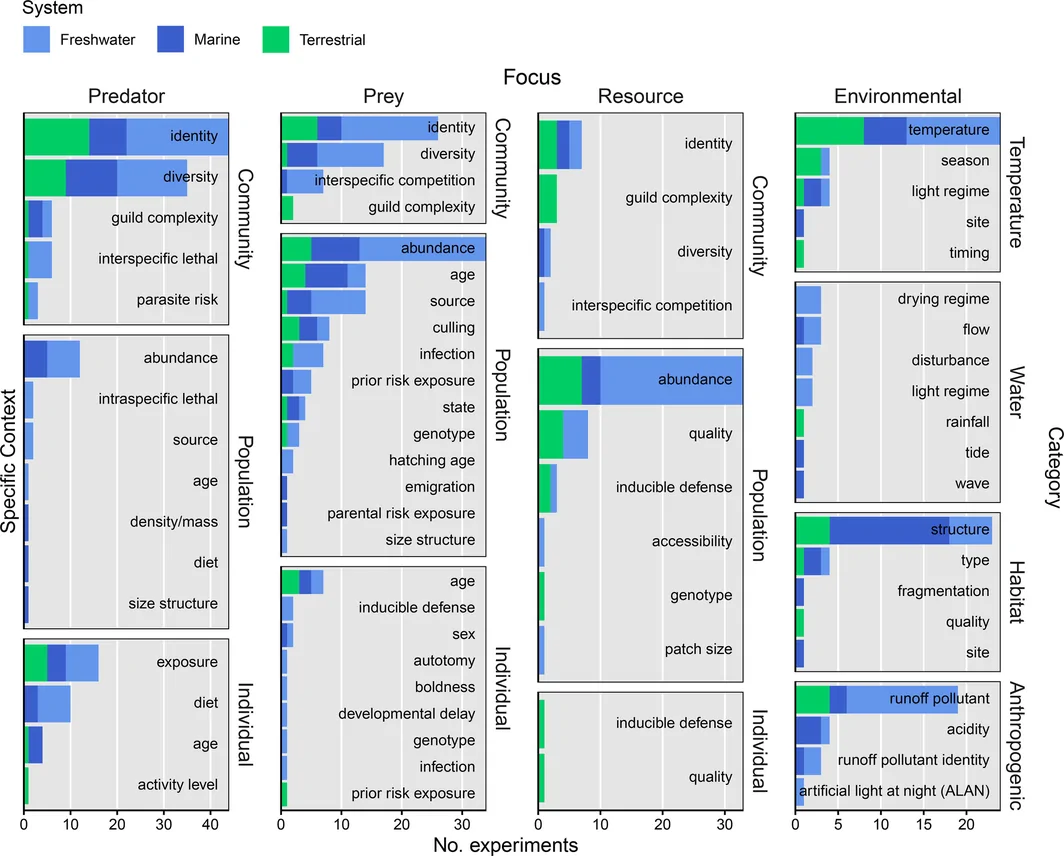
Histograms of the number of publications conducted for each Specific Context within each Focus and Category. Bar colors indicate the System in which the experiments were performed. See Appendix S1: Table S1 for description of each Specific Context.

Among the four Foci, predators (30% of experiments) and prey (33%) were manipulated more than resources (14%) and environment (23%; Table 1, Figure 2). Meanwhile, the number of unique Specific Contexts examined about prey and the environment is greater than the number of unique contexts about predators and resources. Most experiments within the predator Focus were conducted in the ‘community’ Category, while most experiments within the resource Focus were conducted in the ‘population’ Category. Within the prey and environment Foci, experiments were more evenly allocated among the different Categories (Table 1, Figure 2). The number of Specific Contexts tested also varied within each Category (Table 1, Figure 3). For example, while nine different Specific Contexts have been experimentally tested in the ‘individual’ Category for prey, only two have been tested in the ‘individual’ Category for resources. Experiments that manipulated the Specific Contexts ‘abundance’ (e.g., predator abundance or resource abundance) and ‘identity’ (e.g., prey species A vs. prey species B) accounted for 46% of the total.

While most publications (65%) investigated only one context, many manipulated multiple biotic or abiotic contexts, either independently or in a factorial design (Figure 2). Of the 119 publications manipulating multiple contexts, 82% (98 publications) examined the interaction between two or more manipulated contexts in a factorial design (Figure 2). The most frequently evaluated interactions were combinations of the predator and prey Foci (22 publications). In contrast, the least frequently examined interactions were those between resources and environmental conditions (0 publications).

We identified seven Specific Contexts that may have been evaluated sufficiently (i.e., ≥20 experiments) to support synthesis: predator identity, predator diversity, prey identity, prey abundance, resource abundance, environmental temperature, and environmental habitat structure (Figure 3; Table 1). Except for the environmental habitat structure context, which was skewed towards marine experiments (61%), 56% of the experiments on the highly studied Specific Contexts occurred in freshwater systems. Another 16 Specific Contexts had between 5 and 20 studies and with some additional research could soon be candidates for synthesis (Figure 3; Table 1). For instance, manipulations of guild complexity within the predator community (i.e. combining multiple predators without all possible monoculture treatments) and abundance within the predator population had slightly fewer than 20 experiments. Meanwhile, the ‘individual’ Category for resources and prey was relatively understudied.

**FIGURE 4:**
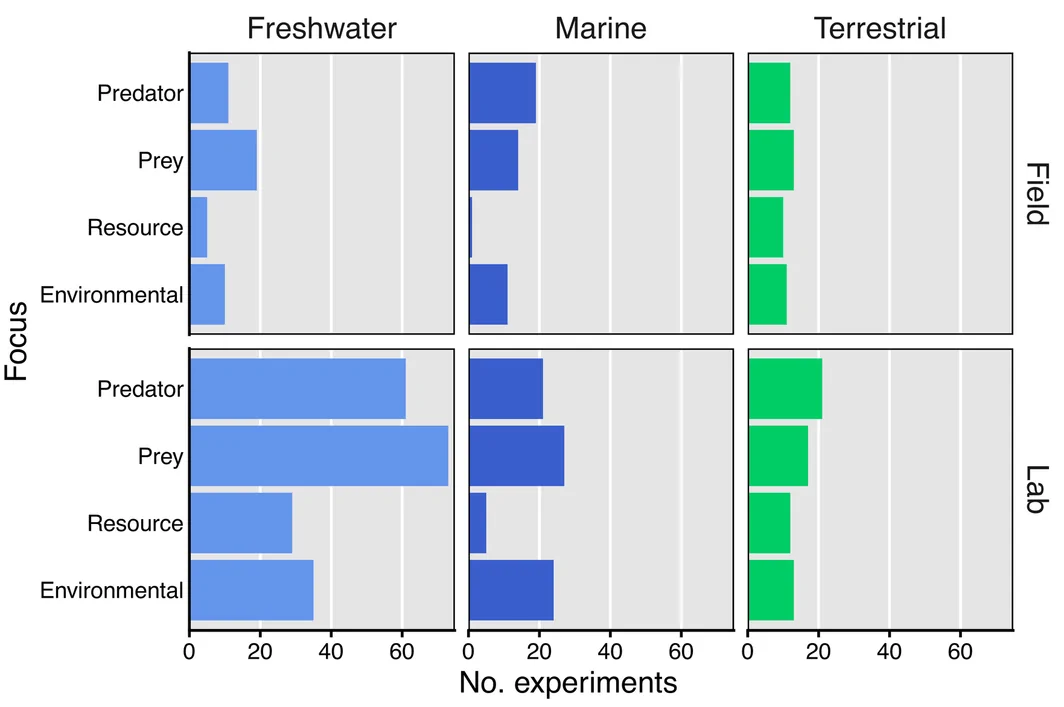
Histograms of the number of experiments on context‐dependent predation risk within each Focus by Venue (lab or field) and System (marine, freshwater, terrestrial).

Among all experiments, 51% were performed in freshwater systems, 24% in marine systems, and 24% in terrestrial systems (Figure 4). Across all systems, 69% of experiments were conducted in laboratory (vs. field) venues, and 41% were conducted in freshwater laboratory settings.

### Highly versus understudied Specific Contexts

**FIGURE 5:**
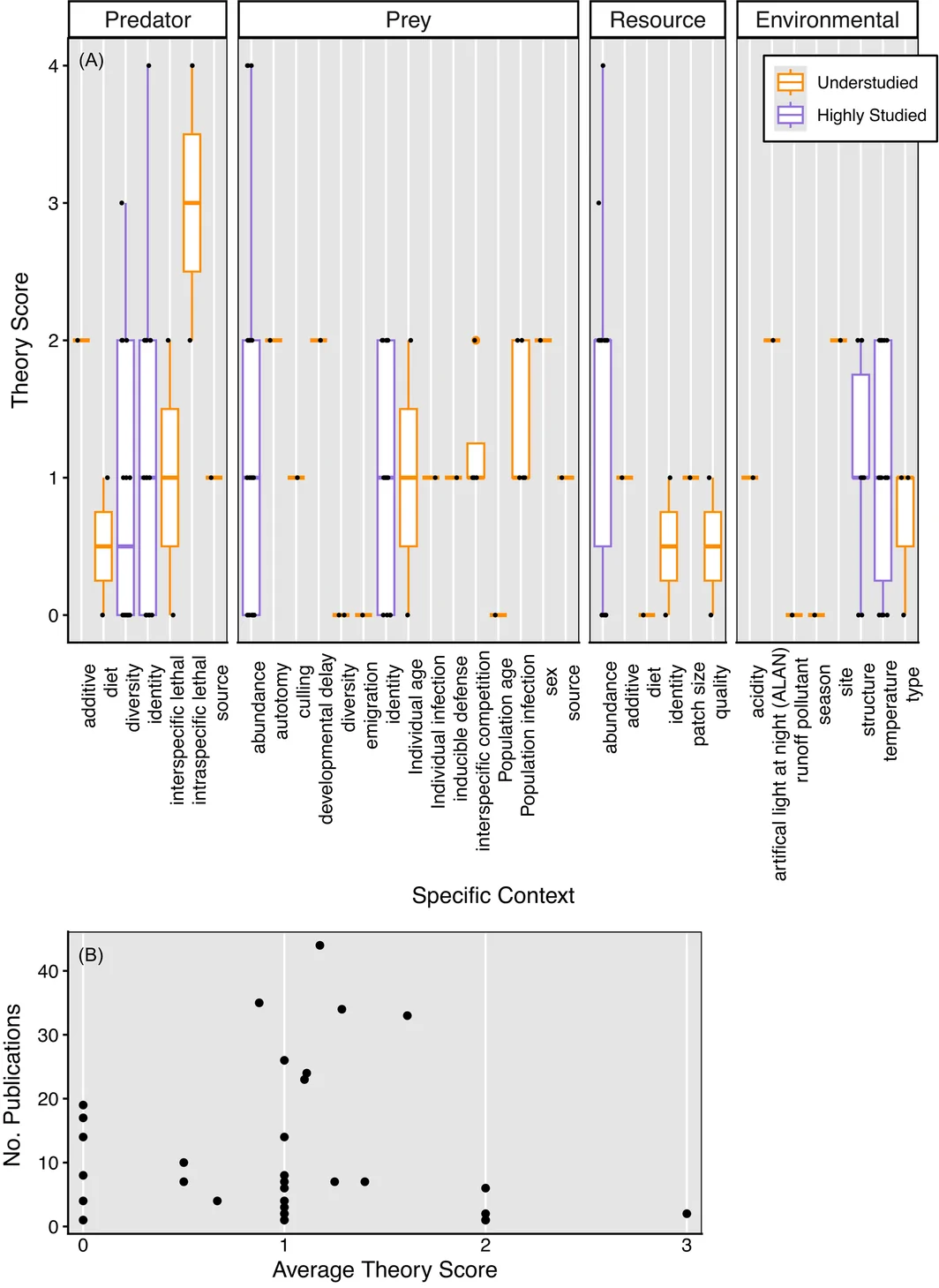
(A) Boxplots displaying the amount of theoretical support (*y*‐axis) in publications for seven highly studied (purple) and 29 understudied (orange) Specific Contexts (*x*‐axis). Specific Contexts are listed alphabetically within each Focus (predator, prey, resource, and environmental). Within each Specific Context, data points represent a randomly selected publication out of total number (*n*) of available publications. (B) Scatter plot displaying the relationship between number of publications within each Specific Context (*y*‐axis) and the average theory score of randomly sampled publications within each Specific Context (*x*‐axis).

Boxplots showed minimal theoretical connectedness of publications within each of the seven highly studied and the 29 understudied Specific Contexts (Figure 5A). In fact, only one understudied (interspecific lethal from the predator Focus) and three highly studied Specific Contexts (predator diversity, prey abundance, and resource abundance) contained a single publication that was strongly connected to theory (i.e., score = 4). We found no support for a significant correlation between average theoretical connectedness and number of publications (*t* = −1.5, df = 35, *p* = 0.15); interestingly, there was a nonsignificant negative trend (Pearson coefficient *r* = −0.24, Figure 5B).

There was also little support for highly studied Specific Contexts being dominated by a small number of co‐author groups. No one co‐author group accounted for more than 13% of publications within a highly studied Specific Context. Overall, there were 30 unique co‐author groups that collectively published 37% (*n* = 80) of the 219 publications about the seven highly studied Specific Contexts.

## DISCUSSION

Our literature review and organizational framework revealed several areas for synthesis and knowledge gaps in the varied literature on context‐dependent predation risk. Unsurprisingly, the protagonists in the predation risk interaction (predator and prey) had their attributes most frequently manipulated, both individually and in factorial combinations. Manipulations of resources (aside from resource abundance) were much less common, as were experiments varying the individual state of prey and resources. Freshwater lab studies made up for more than a third of the experiments.

Manipulations of contexts in the predator and prey Foci were most common, and there were sufficient experiments to pursue synthesis on three Specific Contexts. Within the ‘community’ Category of these two Foci, the substitutive replacement experimental design has been frequently used to test the influence of predator species identity (e.g., active hunting vs. sit‐and‐wait predatory spider species; Schmitz, 2008) or prey species identity (e.g., herbivorous vs. carnivorous snails; Trussell et al., 2003) on risk effects. Examining how traits of different species of predators (e.g., hunting mode) and prey (e.g., anti‐predator behavior) influence predation risk effects would be an interesting area for meta‐analytic synthesis. The biodiversity‐ecosystem‐function design with all monoculture and polyculture treatments has also frequently been used (Stachowicz et al., 2008). Within the ‘population’ Category of the prey Focus, experiments have also frequently tested the influence of prey abundance. Given the rich research on consumer functional responses (Arditi & Ginzburg, 2012; Novak & Stouffer, 2021), a meta‐analytical query could evaluate how varying prey abundance alters predation risk effects. For example, do prey consistently display linear (Type I) or nonlinear (Type II–III) trait responses to risk with variation in the number of conspecific “eyes” (Roberts, 1996), and if so, do such trait responses create functionally similar or dissimilar NCEs and TMIEs?

In the overall less commonly studied Foci of resources and environment, three Specific Contexts were more highly represented. Specifically, in the ‘population’ Category of the former, experiments frequently manipulated the abundance of food resources available to prey in the presence and absence of risk. Meanwhile, in the ‘temperature’ Category of the latter, the NCEs and TMIEs of predators were frequently tested in ambient versus experimentally warmed temperatures. Finally, within Focus/Category of environmental habitat, researchers have experimentally varied the amount of habitat structure available for prey. Because experiments have demonstrated that elevated resources, temperature, and structure can all weaken (Green, 2012; Han et al., 2020; Lord et al., 2017) and strengthen (Babbitt, 2001; Griffen & Byers, 2006; Miller et al., 2014) predation risk effects, meta‐analytic queries would also help resolve discrepancies about these Specific Contexts.

Our framework highlights knowledge gaps that are notable in five different ways. First, the variety of Specific Contexts differs among Foci and Categories. For instance, within the resource Focus, the individual Category has a much lower diversity of unique Specific Contexts compared to the individual Categories of the predator and prey Foci. By highlighting successfully studied areas in one Focus that are not represented in other Foci, we identify novel Specific Contexts yet to be explored. Next, over 86% of the existing Specific Contexts are very understudied, with five or fewer publications. Thus, any of these less represented contexts would benefit from increased research effort. The sparsely populated realm of interactive Specific Contexts also represents fertile ground for experimental results. For example, the effect of pesticide concentration depended on prey identity, with higher pesticides drastically increasing mortality (the NCE) in some amphibian prey species but not others (Relyea, 2003). Fourth, this framework also highlights Specific Contexts requiring only slightly more research before synthesis. For instance, while identity has been well studied in the predator and prey Foci, it has been tested fewer than 10 times in the resource Focus. Similarly, while the prey Focus features many studies on abundance, this Specific Context in the predator Focus would benefit from more research. As increasing predator abundance has been shown to both facilitate and inhibit multiple predator effects on consumption of prey (survival, Sih et al., 1998), further research could elucidate how predator abundance consistently alters NCEs and TMIEs. Finally, none of the Specific Contexts have been examined sufficiently in freshwater, marine, and terrestrial ecosystems, even though ecologists generally acknowledge the importance of cross‐system variation in ecological dynamics (Shurin et al., 2002).

We explored two possible reasons for the disparity in research effort on different Specific Contexts. However, we found no evidence that Specific Contexts with more publications were better motivated by theory, or the result of a small number of coauthor groups publishing on the same topic. Another possible explanation is that individual empirical publications or reviews could have attracted and concentrated research on a few Specific Contexts (e.g., Schmitz et al., 2004 on predator identity/hunting mode). However, this is difficult to test with existing literature search tools (which can tell you whether, but not why a certain paper was cited) and is beyond the scope of our current review. We suspect the most likely reason certain Specific Contexts are highly studied is because they represent general facets of food webs that are shared across many ecosystems. For instance, the abundance of predator, prey, and resource species involved in the risk dynamic are quite general, as are the traits and diversity of these species (predator and prey identity and diversity). In addition, all food webs are being influenced by climate change, and thus it is not surprising that temperature is a frequently studied context. Conversely, the infrequently studied Specific Contexts may represent conditions important to only a few food webs. For example, salinization of lakes due to road salt and wave exposure on rocky intertidal communities are narrower Specific Contexts that are not applicable to all ecosystems.

Given the imbalanced literature revealed by this review, how should the risk effects field strategically address the wide diversity of knowledge gaps? If we assume that the goals of ecology are to test theory and to inform management and societal needs, then we suggest several non‐mutually exclusive guidelines that could be used to identify optimal research areas. Useful contexts to study experimentally might (1) improve connectedness and generalizability by examining response variables explicitly linked to theoretical model predictions and parameters; (2) be manipulable in a manner that represents the continuum of observed natural variation, rather than presence/absence; (3) be able to be ground‐truthed in observational data, such as that collected in long‐term monitoring programs; and (4) be relevant to societal needs such as agriculture, pest control, and fisheries.

As ecologists begin to pursue the understudied Specific Contexts, our organizational framework highlights the need for more field studies and more studies outside of freshwater food webs. Half of the experiments in our database were within freshwater systems, which represent <1% of Earth's surface. Across all systems, laboratory experiments have also been more common than field experiments, particularly for predator and prey manipulations, perhaps for logistical reasons. Given fundamental differences in physical conditions and ecological dynamics among terrestrial, freshwater, and marine systems (Shurin et al., 2002), and that laboratory populations are not always a good analog for interactions occurring in nature (Werner, 1998), there is a definite need for more field experiments and more experiments in terrestrial and marine settings.

In addition to its strengths, our organizational framework and literature review has two limitations to consider. First, we focused solely on laboratory and highly controlled field experiments, when in fact many ecological processes operate at scales larger than the typical replicate of an experimental field plot (Diamond, 1983; Werner, 1998), and it is often logistically or ethically impossible to orthogonally manipulate predation risk at relevant scales. A partial solution is the comparative experimental approach (CEA), where risk treatments are replicated across known spatial/temporal gradients in predator, prey, resource, or environmental conditions (Menge et al., 2003). For example, predator risk cues are often manipulated in field settings, such as marine predatory crab cues at different levels of water turbulence (Large et al., 2011) or predator vocalizations at open versus closed habitat structure (Epperly et al., 2021). This promising approach was used only in 2.5% of published experiments in this review. A second limitation was the logistical difficulty of engaging with a static framework in a dynamic and expanding literature. In fact, the labor involved in this un‐automated review along with the iteratively helpful peer‐review process has rendered this review 5 years behind the existing literature. Undoubtedly, the framework will need to evolve with the literature over time. Developing an interactive visualization allowing the hierarchy of classifications to be explored at multiple resolutions, with links to individual studies and abstracts, could further increase the utility of the database to ecologists. Integrating experiments with observation and mathematical modeling will help to expand the scale of investigations and test their relevance within larger ecosystems (Werner, 1998).

The study of predation risk effects is a growing topic in ecology with implications for the conservation and management of natural resources (Peacor et al., 2022). Through studies of context‐dependence, we can better understand why the NCEs and TMIEs vary in magnitude and direction over space and time. Because many important contexts such as predator identity, diversity, and habitat structure are being altered by anthropogenic actions, this understanding may aid in the prediction of emerging dynamics. Rapid synthesis and informed targeting of new manipulations will optimize the integration of experimental approaches with ongoing observational and modeling approaches to further the development and application of predation risk theory. Much remains for early career and seasoned experimental ecologists to pursue.

## AUTHOR CONTRIBUTIONS

Nicole E. Peckham and David L. Kimbro conceived the study, with Scott D. Peacor significantly shaping methodology. All authors processed papers from 1990 to 2018. Nicole E. Peckham and David L. Kimbro wrote the first draft with consultation from Michael J. Sheriff and Justine A. Smith. Tanya L. Rogers created the figures and aggressively edited drafts. All authors contributed to subsequent drafts.

## CONFLICT OF INTEREST STATEMENT

The authors declare no conflicts of interest.

## Supporting information


Appendix S1.

## Data Availability

Data (Peckham, 2026) are available in the Northeastern University Digital Repository Service at https://hdl.handle.net/2047/D20861766.

## References

[ecy70493-bib-0001] Abrams, P. A. 2008. “Measuring the Impact of Dynamic Antipredator Traits on Predator‐Prey‐Resource Interactions.” Ecology 89: 1640–1649. 18589528 10.1890/07-0764.1

[ecy70493-bib-0002] Arditi, R. , and L. Ginzburg . 2012. How Species Interact: Altering the Standard View on Trophic Ecology. New York: Oxford University Press.

[ecy70493-bib-0003] Babbitt, K. J. 2001. “Behaviour and Growth of Southern Leopard Frog (*Rana sphenocephala*) Tadpoles: Effects of Food and Predation Risk.” Canadian Journal of Zoology 79: 809–814.

[ecy70493-bib-0004] Bar‐On, Y. M. , R. Phillips , and R. Milo . 2018. “The Biomass Distribution on Earth.” Proceedings of the National Academy of Sciences of the United States of America 115: 6506–6511. 29784790 10.1073/pnas.1711842115 PMC6016768

[ecy70493-bib-0005] Bolnick, D. I. , and E. L. Preisser . 2005. “Resource Competition Modifies the Strength of Trait‐Mediated Predator–Prey Interactions: A Meta‐Analysis.” Ecology 86: 2771–2779.

[ecy70493-bib-0006] Chamberlain, S. A. , J. L. Bronstein , and J. A. Rudgers . 2014. “How Context Dependent Are Species Interactions?” Ecology Letters 17: 881–890. 24735225 10.1111/ele.12279

[ecy70493-bib-0007] Diamond, J. M. 1983. “Ecology: Laboratory, Field, and Natural Experiments.” Nature 304: 586–587.

[ecy70493-bib-0008] Epperly, H. K. , M. Clinchy , L. Y. Zanette , and R. A. McCleery . 2021. “Fear of Large Carnivores is Tied to Ungulate Habitat Use: Evidence from a Bifactorial Experiment.” Scientific Reports 11: 12979. 34155290 10.1038/s41598-021-92469-5 PMC8217516

[ecy70493-bib-0009] Green, L. A. 2012. “Refuge Availability Increases Kelp Consumption by Purple Sea Urchins Exposed to Predation Risk Cue.” Aquatic Biology 17: 141–144.

[ecy70493-bib-0010] Griffen, B. D. , and J. E. Byers . 2006. “Partitioning Mechanisms of Predator Interference in Different Habitats.” Oecologia 146: 608–614. 16086166 10.1007/s00442-005-0211-4

[ecy70493-bib-0011] Han, P. , C. Becker , J. Le Bot , R. Larbat , A.‐V. Lavoir , and N. Desneux . 2020. “Plant Nutrient Supply Alters the Magnitude of Indirect Interactions between Insect Herbivores: From Foliar Chemistry to Community Dynamics.” The Journal of Ecology 108: 1497–1510.

[ecy70493-bib-0012] Large, S. I. , D. L. Smee , and G. C. Trussell . 2011. “Environmental Conditions Influence the Frequency of Prey Responses to Predation Risk.” Marine Ecology Progress Series 422: 41–49.

[ecy70493-bib-0013] Lima, S. L. , and L. M. Dill . 1990. “Behavioral Decisions Made Under the Risk of Predation: A Review and Prospectus.” Canadian Journal of Zoology 68: 619–640.

[ecy70493-bib-0014] Lord, J. P. , J. P. Barry , and D. Graves . 2017. “Impact of Climate Change on Direct and Indirect Species Interactions.” Marine Ecology Progress Series 571: 1–11.

[ecy70493-bib-0015] Menge, B. A. , J. Lubchenco , M. E. S. Bracken , F. Chan , M. M. Foley , T. L. Freidenburg , S. D. Gaines , et al. 2003. “Coastal Oceanography Sets the Pace of Rocky Intertidal Community Dynamics.” Proceedings of the National Academy of Sciences of the United States of America 100: 12229–12234. 14512513 10.1073/pnas.1534875100 PMC218741

[ecy70493-bib-0016] Miller, L. P. , C. M. Matassa , and G. C. Trussell . 2014. “Climate Change Enhances the Negative Effects of Predation Risk on an Intermediate Consumer.” Global Change Biology 20: 3834–3844. 24947942 10.1111/gcb.12639

[ecy70493-bib-0017] Novak, M. , and D. B. Stouffer . 2021. “Systematic Bias in Studies of Consumer Functional Responses.” Ecology Letters 24(3): 580–593. 33381898 10.1111/ele.13660

[ecy70493-bib-0018] Orrock, J. L. , E. L. Preisser , J. H. Grabowski , and G. C. Trussell . 2013. “The Cost of Safety: Refuges Increase the Impact of Predation Risk in Aquatic Systems.” Ecology 94: 573–579. 23687883 10.1890/12-0502.1

[ecy70493-bib-0019] Peacor, S. D. , B. T. Barton , D. L. Kimbro , A. Sih , and M. J. Sheriff . 2020. “A Framework and Standardized Terminology to Facilitate the Study of Predation‐Risk Effects.” Ecology 101: e03152. 32736416 10.1002/ecy.3152

[ecy70493-bib-0020] Peacor, S. D. , N. J. Dorn , J. A. Smith , N. E. Peckham , M. J. Cherry , M. J. Sheriff , and D. L. Kimbro . 2022. “A Skewed Literature: Few Studies Evaluate the Contribution of Predation‐Risk Effects to Natural Field Patterns.” Ecology Letters 25: 2048–2061. 35925978 10.1111/ele.14075 PMC9545701

[ecy70493-bib-0021] Peacor, S. D. , C. Song , J. R. Bence , A. A. Briggs , E. A. Hamman , and C. W. Osenberg . 2025. “Ecological Meta‐Analyses Often Produce Unwarranted Results.” Ecology 106: e70269. 41358409 10.1002/ecy.70269 PMC12683612

[ecy70493-bib-0022] Peckarsky, B. L. , P. A. Abrams , D. I. Bolnick , L. M. Dill , J. H. Grabowski , B. Luttbeg , J. L. Orrock , et al. 2008. “Revisiting the Classics: Considering Nonconsumptive Effects in Textbook Examples of Predator‐Prey Interactions.” Ecology 89: 2416–2425. 18831163 10.1890/07-1131.1

[ecy70493-bib-0023] Peckham, N. 2026. “Knowledge Gaps and Areas for Synthesis in Experimental Studies of Context‐Dependent Predation Risk.” Dataset. Northeastern University Digital Repository Service. https://hdl.handle.net/2047/D20861766 10.1002/ecy.7049342710508

[ecy70493-bib-0024] Preisser, E. L. , D. I. Bolnick , and J. H. Grabowski . 2009. “Resource Dynamics Influence the Strength of Non‐Consumptive Predator Effects on Prey.” Ecology Letters 12: 315–323. 19243407 10.1111/j.1461-0248.2009.01290.x

[ecy70493-bib-0025] Preisser, E. L. , J. L. Orrock , and O. J. Schmitz . 2007. “Predator Hunting Mode and Habitat Domain Alter Nonconsumptive Effects in Predator‐Prey Interactions.” Ecology 88: 2744–2751. 18051642 10.1890/07-0260.1

[ecy70493-bib-0026] Relyea, R. A. 2003. “Predator Cues and Pesticides: A Double Dose of Danger for Amphibians.” Ecological applications: a publication of the Ecological Society of America 13: 1515–1521.

[ecy70493-bib-0027] Roberts, G. 1996. “Why Individual Vigilance Declines as Group Size Increases.” Animal Behaviour 51(5): 1077–1086.

[ecy70493-bib-0028] Schmitz, O. J. 2008. “Effects of Predator Hunting Mode on Grassland Ecosystem Function.” Science 319: 952–954. 18276890 10.1126/science.1152355

[ecy70493-bib-0029] Schmitz, O. J. , V. Krivan , and O. Ovadia . 2004. “Trophic Cascades: The Primacy of Trait‐Mediated Indirect Interactions: Primacy of Trait‐Mediated Indirect Interactions.” Ecology Letters 7: 153–163.

[ecy70493-bib-0030] Shurin, J. B. , E. T. Borer , E. W. Seabloom , K. Anderson , C. A. Blanchette , B. Broitman , S. D. Cooper , and B. S. Halpern . 2002. “A Cross‐Ecosystem Comparison of the Strength of Trophic Cascades.” Ecology Letters 5: 785–791.

[ecy70493-bib-0031] Sih, A. , G. Englund , and D. Wooster . 1998. “Emergent Impacts of Multiple Predators on Prey.” Trends in Ecology & Evolution 13: 350–355. 21238339 10.1016/s0169-5347(98)01437-2

[ecy70493-bib-0032] Stachowicz, J. J. , R. J. Best , M. E. S. Bracken , and M. H. Graham . 2008. “Complementarity in Marine Biodiversity Manipulations: Reconciling Divergent Evidence from Field and Mesocosm Experiments.” Proceedings of the National Academy of Sciences of the United States of America 105: 18842–18847. 19028868 10.1073/pnas.0806425105 PMC2596222

[ecy70493-bib-0033] Trussell, G. C. , P. J. Ewanchuk , and M. D. Bertness . 2003. “Trait‐Mediated Effects in Rocky Intertidal Food Chains: Predator Risk Cues Alter Prey Feeding Rates.” Ecology 84: 629–640.

[ecy70493-bib-0034] Werner, E. E. 1998. “Ecological Experiments and a Research Program in Community Ecology.” In Experimental Ecology: Issues and Perspectives, edited by W. J. R. Bernardo , 1–18. Oxford: Oxford University Press.

[ecy70493-bib-0035] Werner, E. E. , and S. D. Peacor . 2003. “A Review of Trait‐Mediated Indirect Interactions in Ecological Communities.” Ecology 84: 1083–1100.

[ecy70493-bib-0036] Wirsing, A. J. , M. R. Heithaus , J. S. Brown , B. P. Kotler , and O. J. Schmitz . 2021. “The Context Dependence of Non‐Consumptive Predator Effects.” Ecology Letters 24: 113–129. 32990363 10.1111/ele.13614

