# Supplementary material for: Knowledge gaps and areas for synthesis in experimental studies of context‐dependent predation risk
Source: Ecology. 2026 Sep 8;107(9):e70493. doi: 10.1002/ecy.70493 (PMC13553125; doi:10.1002/ecy.70493)
Supplement: Supplementary file 1 [file ECY-107-e70493-s001.pdf]

Appendix S1

Knowledge gaps and areas for synthesis in experimental studies of context-dependent predation risk

Nicole E. Peckham, Scott D. Peacor, Michael J. Sheriff, Justine A. Smith, Nathan J. Dorn, Michael J. Cherry, Tanya L. Rogers, and David L. Kimbro

Ecology

Table S1: Summary list of relevant Foci, Categories, and Specific Contexts. Each Specific Context is associated with a methodological description and a generalized example of experimental treatments.

**Foci**	**Categories**	**Specific Context**	**Experimental manipulation**	**Example of experimental treatments**
resource, prey, predator	population	abundance	number of individuals at beginning of experiment	10, 20, 30 individuals
environmental	anthropogenic	acidity	acidity (pH) of water (fresh or saltwater) by adding CO_2	ambient versus elevated CO_2 levels
predator	individual	activity level	activity level of the predator	low activity level score versus high activity level score
prey, predator	population, individual	age	age (or stage or size) of organisms	adult versus juvenile
environmental	anthropogenic	artificial light at night (ALAN)	ALAN created with outdoor LED flood light	ALAN present versus ALAN absent
prey	individual	autotomy	organisms who lost limbs during previous predator encounter	appendages present versus absent
prey	individual	boldness	organismal personality determined by previous behavioral assay (e.g., emergence time in novel environment)	shy versus bold individual of same species
prey	population	culling	decreasing the abundance of organisms after the experiment begins to simulate consumptive effect of predator	6% removal versus no removal
predator	individual	density/mass	the size and density of individuals in an experiment while biomass is held constant	one large, few medium, many small

prey	individual	developmental delay	prey development “arrested” by reducing temperature	delayed prey versus nondelayed prey
predator	individual, population	diet	predators fed differing diets	risk cues of predator consuming diet A versus diet B
environmental	water	disturbance	hand tumbled substrate to simulate flood	hand tumbling treatment present versus absent
prey, predator, resource	community	diversity	species A looked at in monoculture, species B looked at in monoculture, and looked at together	species A versus species B versus species A+B
environmental	water	drying regime	the amount of drying done in an experiment	fast drying ponds, slow drying ponds, drying absent
prey	individual	emigration	ability of prey to leave risky area	emigration present versus absent
predator	individual	exposure	duration or magnitude of exposure to cue (actual cue or simulated)	strong simulated attack (6 pinches) versus weak simulated attack (2 pinches)
environmental	water	flow	velocity of water	10 cm/s versus 25 cm/s
environmental	habitat	fragmentation	amount of fragmentation of habitat	1 large habitat versus 2 small habitats
prey, resource	individual, population	genotype	genotype within species	genotype A versus genotype B
predator, resource, prey	community	guild complexity	species A examined in monoculture treatment and in treatment with species B, but species B not examined in monoculture treatment	species A, species A+B, species A+B+C
prey	population	hatching age	amount of time spent in egg before hatching	4 days versus 6 days
predator, resource, prey	community	identity	multiple species looked at individually	species A versus species B
prey, resource	individual, population	inducible defense	individuals displaying induced defense	defended morph versus undefended morph
prey	individual, population	infection	infection state of prey	infected prey versus uninfected prey
prey, resource	community	interspecific competition	prey in competition with other species (fitness response in other species not quantified)	prey species alone versus prey species with other species as competitors

predator	community	interspecific lethal	prey exposure to a lethal predator of a different species than prey	prey individuals alone versus prey individuals with lethal predator of different species
predator	population	intraspecific lethal	prey exposure to a lethal predator of the same species as prey	prey individuals alone versus prey individuals with lethal cannibal
environmental	water, temperature	light regime	amount of light organisms are exposed to	shade present versus shade absent
predator	community	parasite risk	the presence of a parasite with the potential to infect prey	virus present versus virus absent
prey	population	parental risk exposure	whether the parents of prey organisms were exposed to risk	offspring of parents exposed to risk versus not exposed to risk
resource	population	patch size	size of resource patch	small patch versus large patch
prey	population, individual	prior predator exposure	prey organisms were previously exposed to predators	naive prey versus prey with previous predator exposure
resource, environmental	population, habitat, individual	quality	quality of the environment or resource	good quality habitat versus poor quality habitat
environmental	water	rainfall	amount of rainfall received	low versus ambient versus high rainfall
resource	population	resource accessibility	how accessible the resources are to prey	periphyton on cobbles versus periphyton on floating mats
environmental	anthropogenic	runoff pollutant identity	type of runoff pollutant	atrazine versus carbaryl versus endosulfan
environmental	anthropogenic	runoff pollutant	presence or concentration of runoff pollutant	insecticide present versus insecticide absent
environmental	temperature	season	either temporal or reproductive cycle	breeding season versus nonbreeding season
prey	individual	sex	sex of prey organisms	male versus female
environmental	temperature, habitat	site	location experiments were conducted	north sites versus south sites
prey, predator	population	size structure	size composition	all large individuals versus mixed large and small
predator, prey	population	source	collection site of organisms	organisms collected from a risky site versus nonrisky site

prey	population	state	the physiological state of the prey	starved prey versus fed prey
environmental	habitat	structure	the relative amount of structure an environment (or resource) had	high refuge versus low refuge
environmental	temperature	temperature	the temperature the experiment took place in	ambient versus +2°C
environmental	water	tide	the tidal regime	site with diurnal tides versus mixed semidiurnal tides
environmental	temperature	timing	when warming occurs	daytime versus nighttime warming
environmental	habitat	type	type of habitat the experiment took place in	oyster reefs versus mudflats
environmental	water	wave	the amount of wave action an experiment received	wave exposed site versus protected site

Table S2: Summary of scoring system (left column) used to evaluate publications on the amount of theoretical development for a Specific Context. In the right column, each score is associated with a general description and a hypothetical example.

Score (0-4)	Description
0	In the Introduction of the paper, the manipulated Specific Context was either not mentioned or was introduced but not connected to any theory about predation risk effects. For instance, "Predation risk affects prey growth. Because nutrients and predators also affect prey growth, we examined ether whether prey growth was affected by an interaction between risk and nutrients."
1	In the Introduction of the paper, the authors developed a simple deductive argument of how a manipulated Specific Context could mechanistically affect the outcome of risk effects; and the mechanism was loosely based on theory. For example, "Theory suggests that the nonconsumptive effect (NCE) of predators on prey growth depends on trade-offs. Because refuge or nutrients will affect those tradeoffs, we expected variation in refuge (or nutrients) to influence the strength of the NCE on prey growth".
2	In the Introduction of the paper, the authors clearly identified a testable hypothesis about the manipulated Specific Context with a mechanistic, rule-of-thumb pathway for how the context would interact with predation risk effects. And the associated experiment was designed to test that mechanistic pathway. For instance, "The "X" hypothesis claims that resource abundance will alter the trade-off prey experience between risk and resource acquisition so that NCE strength will be minimized in resource rich versus poor conditions. To test this hypothesis, we…"
3	The Introduction of the publication presented a hypothesis of how the Specific Context would interact with predation risk effects by using a mathematical or conceptual model. Then, the experiment of the publication was designed to test the influence of the Specific Context according to the theory and model. For instance, in Peacor (2002), the authors modified existing theory to evaluate the influence of resource parameters on resource feedback pathways to modify the strength of NCEs. A figure was used to illustrate the different predicted outcomes and the experimental design aligned with those predictions.
4	The Introduction of the publication presented a hypothesis based on a model developed for the featured study system. The mathematical model was used and the parameters of the model were based on the featured study system and/or experimental results from the publication.

References:

Peacor, S. D. 2002. Positive effect of predators on prey growth rate through induced modifications of prey behaviour. Ecology Letters 5:77–85.
